# Compassion satisfaction and compassion fatigue in humanitarian aid workers: the relationship with shared trauma and coping mechanisms

**DOI:** 10.3389/fpsyg.2025.1522092

**Published:** 2025-04-02

**Authors:** Mohamed Adwi, Nada Abdellatif, Ismail Sadek, Mohamed Elsheikh

**Affiliations:** ^1^Research and Development Department, Shezlong Inc., Giza, Egypt; ^2^Department of Psychiatry, Faculty of Medicine, Al-Azhar University, Cairo, Egypt

**Keywords:** burnout, compassion satisfaction, displacement, humanitarian aid work, shared trauma, secondary traumatic stress, Middle East

## Abstract

**Introduction:**

Forced displacement constitutes a global crisis impacting millions of people especially in the Middle East, leaving them impacted by traumatic history. Humanitarian aid workers (HAWs) who support displaced individuals are exposed to high risk of burnout and secondary traumatic stress (STS).

**Methods:**

This study aimed to identify the prevalence of compassion satisfaction (CS) and compassion fatigue (CF), referring to burnout and STS, respectively, using the Professional Quality of Life Scale (ProQOL). The study explored the relationships between these factors and personal variables that are related to shared trauma, as well as coping mechanisms assessed using the Brief-COPE questionnaire among Middle Eastern HAWs working with displaced individuals.

**Results:**

The study involved 78 HAWs supporting displaced individuals in the Middle East. The mean age was 25.81 years (SD = ± 5.54); 55% were females, and the majority (88%) were Syrians. Approximately 90% of participants were engaged in Turkey and Syria. The most prevalent coping mechanisms were religion and planning. Being a graduate predicted burnout, whereas older age, previous mental diagnosis, and shared trauma predicted higher STS levels. Compassion satisfaction was predicted by active coping, and compassion fatigue was predicted by negative coping.

**Conclusion:**

HAWs require education to recognize CF signs and psychological training to promote effective coping mechanisms, mitigate CF, and enhance higher levels of CS. More research is needed on the psychology of HAWs and the role of shared trauma and coping mechanisms.

## Introduction

Forced displacement is an escalating global phenomenon that disrupts the lives of millions of individuals and communities, profoundly impacting their wellbeing and stability. In 2023, the United Nations High Commissioner for Refugees (UNHCR) estimated that more than one in 73 people worldwide were forcibly displaced, totalling approximately 110 million globally, and this number is expected to increase to 130.8 million (UNHCR, 2024; UNHCR, 2023). Individuals often face displacement due to exposure to a diverse array of traumatic events that jeopardize their safety, including torture, violence, kidnapping, and the loss of loved ones, compelling them to seek essential needs outside their homes. Even if they depart from their traumatizing homes, they carry the burden of trauma with them, which adds new layers of distress to the challenges of relocating and adapting to new circumstances (Henry, 2012; Theisen-Womersley, 2021). Moreover, the departure trajectory itself presents inherent risks, encompassing potential threats such as trafficking, forced labor, and the risk of financial or sexual exploitation (IOM, 2024).

In this context, the role of humanitarian aid workers (HAWs) is paramount because they provide vital support and assistance for displaced individuals and offer various resources to help alleviate their suffering. The occupational duties of HAWs involve conducting needs assessments and formulating plans based on individuals’ vulnerabilities, which requires hearing the traumatic details and challenges faced by their clients. This necessitates empathy and the provision of care for the clients, especially in times of crisis (Walkup, 1997). In addition, HAWs might operate in environments marked by risks related to political instability, poverty, and natural disasters, which could entail the risk of death, injury, or witnessing violence against loved ones (Curling and Simmons, 2010). These environments pose threats of death or injury, and the potential for witnessing violence against loved ones. This reality implies that Humanitarian Aid Workers (HAWs), as trauma helpers, may themselves become trauma survivors as they both suffer from the same collective trauma. The dual exposure to trauma at the personal and professional level is often referred to as shared trauma (Tosone, 2011).

This challenging environment in which humanitarian aid workers (HAWs) operate, combined with their continual exposure to traumatized casualties, makes them vulnerable to the development of various psychological disorders (Lopes Cardozo et al., 2012). Research has shown that HAWs have a greater prevalence of depression, anxiety and posttraumatic stress disorder (PTSD), compared to the general population (Strohmeier and Scholte, 2015).

Additionally, increasing attention has been directed toward the prevalence of secondary traumatic stress and burnout among HAWs (Apostolidou, 2016; Jachens et al., 2016; Jones and Williamson, 2014). Secondary traumatic stress (STS), also known as vicarious trauma, was initially introduced in 1990 by McCann and Pearlman as a framework for understanding the response to working with traumatized individuals (McCann and Pearlman, 1990). STS entails cognitive, behavioral, and emotional responses due to indirect exposure to trauma, which elicits responses similar to those observed in PTSD patients. These shared symptoms include hyperarousal, re-experiencing the traumatic events, social withdrawal, depression, and anxiety (Akinsulure-Smith et al., 2018; Connorton et al., 2012; Figley, 2002). Burnout is referred to in the International Classification of Diseases 11th Revision (ICD-11) as “a syndrome conceptualized as resulting from chronic workplace stress that has not been successfully managed. It is characterized by three dimensions: (1) feelings of energy depletion or exhaustion; (2) increased mental distance from one’s job or feelings of negativism or cynicism related to one’s job; and (3) a sense of ineffectiveness and lack of accomplishment” (WHO, 2019). Both STS and burnout are regarded as negative consequences of working in the humanitarian aid field.

Occupation-related psychological consequences have been the subject of research interest, and multiple frameworks have been proposed. In the context of aid work, Stamm (Ano and Vasconcelles, 2005; Ozcan et al., 2021; Stamm, 2009) conceptualized professional quality of life as a construct encompassing both positive and negative aspects of professions involving caregiving. Compassion fatigue (CF) comprises two subdomains, namely, STS and burnout, while compassion satisfaction (CS) refers to the feelings of pleasure and meaning derived from providing care (Geiling et al., 2022; Sagaltici et al., 2022).

The levels of CF and CS among HAWs have been demonstrated to vary widely across studies, as they are influenced by various factors, including the work environment, client factors, and personal characteristics (Roberts et al., 2021). Some personal factors related to age, sex, and previous mental diagnosis could influence the levels of CF and CS (Ager et al., 2012). In addition, shared trauma, in which victims and aid workers experience a traumatic event, may play a pivotal role (Hamid et al., 2020). Moreover, the variations in the levels could be explained in part by the adoption of different coping mechanisms. Coping mechanisms refer to adjusting cognitive and behavioral responses to fulfil challenging demands or stressful events and are considered one of the main factors in stress management (Collins, 2008; Folkman and Moskowitz, 2004). Some coping mechanisms are deemed positive and linked to positive mental outcomes, while maladaptive coping mechanisms might accelerate burnout and STS (Kirby et al., 2011; Maresca et al., 2022).

It is crucial for aid organizations and individuals to understand these factors to enhance CS and mitigate CF. Unaddressed CF among HAWs can lead to negative consequences, including a decline in overall quality of life, diminished work performance, strained social relationships, reduced empathy toward care recipients, and feelings of withdrawal and isolation (Adams et al., 2006). To our knowledge, there is limited research on the relationship between professional quality of life, coping mechanisms, and shared trauma among humanitarian aid workers (HAWs) supporting displaced individuals (Foo et al., 2023; Young et al., 2018). Therefore, this preliminary study aims to identify different factors associated with CF and CS and their relationship with different coping mechanisms as a step toward building supportive recommendations for HAWs aiding displaced individuals specifically and aid workers overall.

## Methods

### Study population and data collection

HAWs work with displaced individuals due to natural and man-made disasters. This study was conducted in January 2024 as part of an online psychoeducational workshop on stress relief, conducted by Shezlong, an Arabic online platform for mental health, to 98 nongovernmental organization (NGO) workers. After the workshops, all the registered participants received an email containing a link to the questionnaire. The email explained the study details and requested consent before proceeding with the questions. After completing the questionnaire, the participants received an automatic email with their scores, interpretations, and additional guidance on stress management tailored to the context of humanitarian aid work. Each participant had the choice to withdraw from the study at any point. The response rate was 79.5%. To minimize potential desirability bias and encourage candid responses, we ensured complete anonymity and confidentiality in the data collection, with no identifying information recorded.

The assessed NGO is a nonprofit humanitarian organization with international reach, primarily operating in Syria, Turkey, Jordan, and Lebanon. While it functions as a national NGO, it has international influence, with a focus on emergency relief efforts, educational support, medical assistance, and shelter programs for displaced individuals. Its financial resources rely on public donations, international crowdfunding campaigns, and community fundraising initiatives. There were no formal mental health support programs for its workers or volunteers. The authors are unable to provide further details about the NGO for reasons of the security of the people involved.

### Study measures

1. Personal characteristics: to measure sex, age, education, marital status, nationality, country of work, nature of work, fieldwork (including contact with displaced individuals, office-based or both), and years of experience. Participants were asked whether they had been previously diagnosed with a mental health condition using a single yes or no question. Individuals were asked about shared trauma using a single yes or no question to determine whether they had experienced an event similar to that experienced by the individuals they assisted. The questions assessing personal characteristics were designed by the authors specifically for this study.
2. The Professional Quality of Life Questionnaire (ProQOL 5) is a 30-item self-report survey that assesses three subscales among helping professionals: CS, burnout, and STS. Example items include “I am preoccupied with more than one person I help” and “I get satisfaction from being able to help people.” Each item is rated on a Likert scale where 1 = never, 2 = rarely, 3 = sometimes, 4 = often, and 5 = very often. For each subscale, the scoring system denotes the level experienced by the worker: 22 or less is low, between 23 and 41 is moderate, and 42 or more is considered high. The translated Arabic versions were used (Stamm, 2010) and had accepted psychometric analysis for CS (Cronbach’s *α* = 0.84), burnout (Cronbach’s α = 0.73), and STS (Cronbach’s α = 0.78) (Hamid et al., 2021).
3. Brief-COPE: a self-reported questionnaire comprising 28 items that is specifically used to evaluate both healthy and unhealthy coping mechanisms employed in response to stressful life events. Each item is scored on a Likert scale ranging from 1 = I have not been doing this at all to 2 = A little bit, 3 = A medium amount, and 4 = I have been doing this a lot. With 2 items for each, 14 coping mechanisms were assessed: active coping, use of informational support, positive reframing, planning, emotional support, venting, humor, acceptance, religion, self-blame, self-distraction, denial, substance use, and behavioral disengagement (Carver, 1997). Example items include “I’ve been criticizing myself.” and “I’ve been looking for something good in what is happening.” The Arabic valid translation tool was used to reveal 3 main subscales based on exploratory factor analysis: active coping, passive coping, and support seeking, with composite reliability scores of 0.84, 0.75, and 0.81, respectively (Alghamdi, 2020). In this study, we evaluated the 14 specific coping mechanisms, and the three general subscales identified in the Arabic translation. The active coping subscale consists of active coping, planning, positive reframing, acceptance, humor, religion, self-distraction, and venting. The passive coping subscale includes the denial, substance use, behavioral disengagement, and self-blame subscales, along with one item each from the self-distraction and venting subscales. Finally, the seeking support subscale included items on emotional and instrumental support.

### Ethical approval

Ethics approval was obtained from the Ethics Committee of Al-Azhar Faculty of Medicine with the registry number of “sych.74 Med.Research.prevalence” Depression/Pts.-MS.0000007. All procedures performed in studies involving human participants were in accordance with the ethical standards of the institutional research committee of Al-Azhar Faculty of Medicine and the 1964 Helsinki Declaration and its later amendments or comparable ethical standards.

### Statistical analysis

The collected data were coded and analysed using SPSS software (Armonk, NY: IBM Corp, version 25). Quantitative variables are presented as the mean ± SD, while the categorical variables are expressed as counts (%). Normality was assessed using the Kolmogorov––Smirnov test (KS). Based on the results of the KS test, the relationships between continuous variables and ProQOL outcomes were examined using independent t-tests, Mann–Whitney tests, one-way ANOVA, and Kruskal-Wallis tests. Pearson correlation analysis was used to investigate the relationship between coping mechanisms and ProQOL. Linear regression models were constructed using significant personal characteristics (Model I), Ghamdi coping strategies (Model II), and 14 coping strategies, with *p* < 0.1 according to the Pearson correlation (Model III). Statistical significance was set at *p* < 0.05.

## Results

### Personal characteristics

The study included 78 participants, 55.1% of whom were female. Participants’ ages ranged from 17 to 36 years, with a mean age of 25.81 years (SD = ± 5.54). In terms of education levels, university students comprised 43.6%, followed by graduates (41.0%) and secondary students (15.4%). Most participants reported never being married (65.4%), while 34.6% were married, including 26 currently married and one divorced. The sample was dominated by Syrian (88.4%), with smaller percentages of Turkish (1.3%), Syrian-Turkish (6.4%), Egyptian (1.3%), and Omani (2.6%). Work locations were primarily in Turkey (61.5%), followed by Syria (28.2%) and other regions (10.3%). Among the participants, 39.7% were exclusively office based, 23.1% engaged in fieldwork with direct contact with displaced individuals, and 37.2% performed both office and field duties. The mean years of experience was 4.31 (SD = 3.3). A minority of participants reported a previous diagnosis of a mental health disorder (12.8%), while a large proportion reported shared traumatic experiences with their clients (83.3%) (Table 1).

**Table 1:** Personal characteristics and professional quality of life.

Study sample (*N* = 78)	*N* (%)
Sex	
Male	35 (44.9)
Female	43 (55.1)
Age	
Min. – Max.	17.0–36.0
Mean ± SD	25.81 ± 5.54
Education	
Secondary student	12 (15.4)
University student	34 (43.6)
Graduate	32 (41.0)
Marital status	
Never married	51 (65.4)
Ever married	27 (34.6)
Nationality	
Syrian	69 (88.4)
Turkish	1 (1.3)
Syrian – Turkish	5 (6.4)
Egyptian	1 (1.3)
Omani	2 (2.6)
Country of work	
Turkey	48 (61.5)
Syria	22 (28.2)
Other	8 (10.3)
Nature of work	
Office work only	31 (39.7)
Field work and direct contact with victims only	18 (23.1)
Both office and field work	29 (37.2)
Years of experience	
Min. – Max.	0.16–12.0
Mean ± SD	4.31 ± 3.3
Previous mental diagnosis	
Yes	10 (12.8)
No	68 (87.2)
Shared Trauma	
Yes	65 (83.3)
No	13 (16.7)
Compassion satisfaction (CS)	
Low	0 (0.0)
Moderate	47 (60.3)
High	31 (39.7)
Min. – Max.	25.0–50.0
Mean ± SD.	39.54 ± 5.88
Burnout	
Low	29 (37.2)
Moderate	49 (62.8)
High	0 (0.0)
Min. – Max.	13.0–37.0
Mean ± SD	24.73 ± 5.25
Secondary traumatic stress (STS)	
Low	18 (23.1)
Moderate	59 (75.6)
High	1 (1.3)
Min. – Max.	11.0–42.0
Mean ± SD	28.31 ± 7.22

### Professional quality of life

Regarding ProQoL of life, none of the participants had low levels of CS. A total of 60.3% reported moderate levels of CS, while 39.7% reported high levels of CS. Regarding burnout, 37.2% exhibited low levels, 62.8% reported moderate levels, and none of the participants reported high levels. In terms of STS, approximately one-quarter of the participants (23.1%) reported low levels, three-quarters (75.6%) reported moderate levels, and only 1.3% reported high levels of STS (Table 1).

### Coping mechanisms

**Figure 1:**
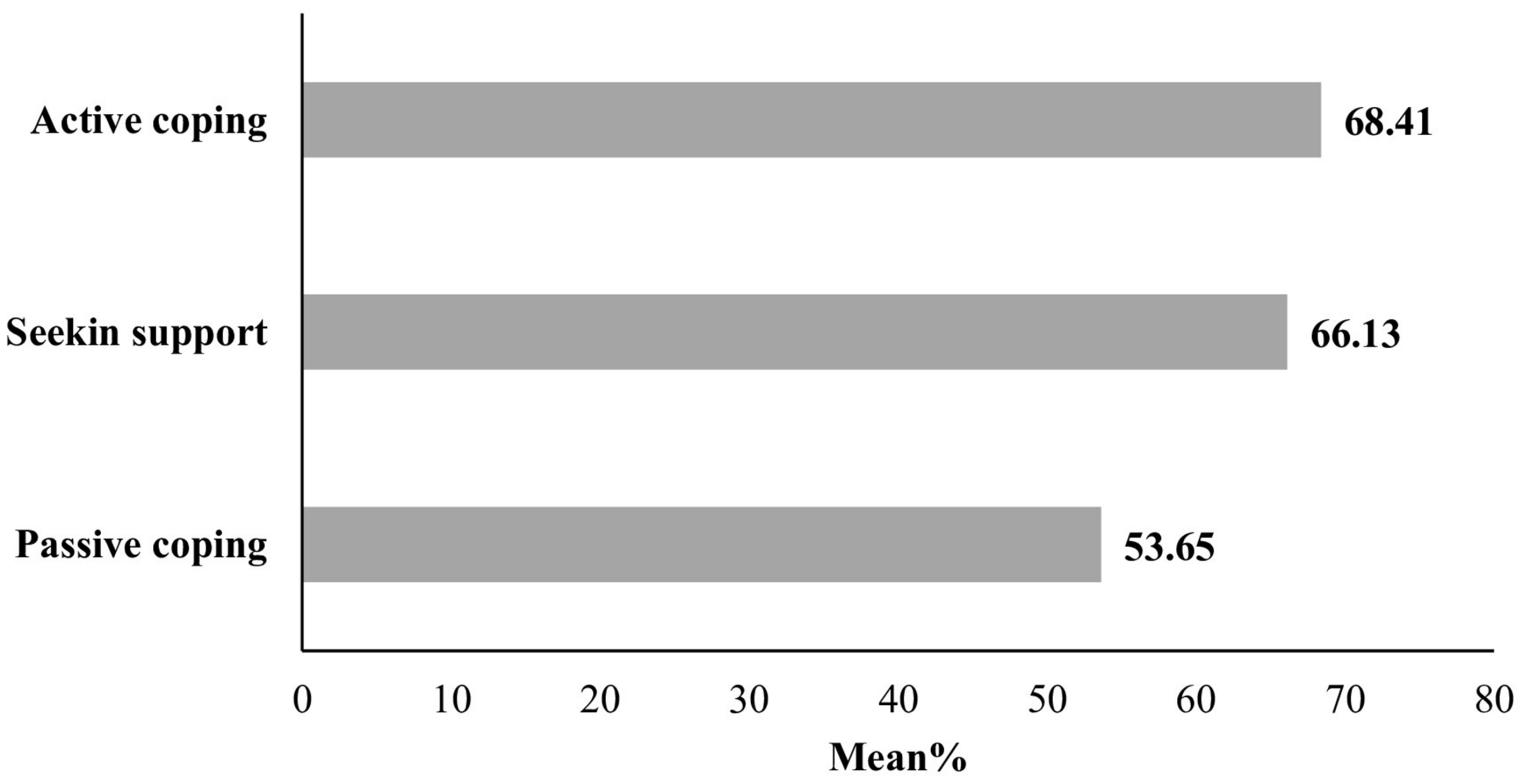
Mean percentage of the general coping mechanisms.

**Figure 2:**
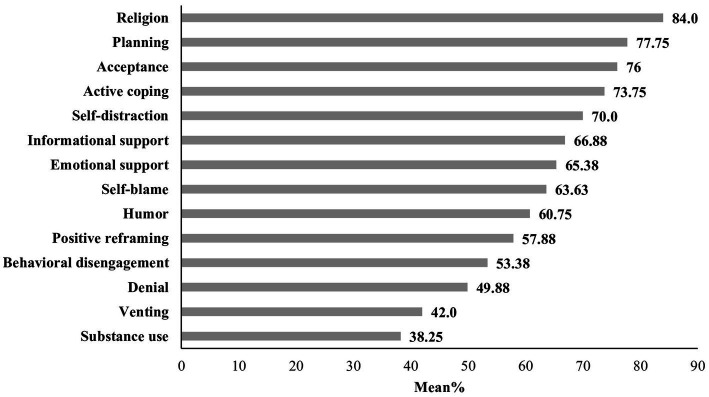
Mean percentage of the specific coping mechanisms.

With respect to the subscales of the general coping mechanism, Figure 1 shows that the most prevalent coping was active coping, with a mean percentage of 68.41%, followed by seeking support (66.13%), while passive coping had a mean percentage of 53.657% (Supplementary Table 1). Among the 14 coping mechanisms analyzed, Figure 2 shows that the leading four were religion (84.0%), planning (77.8%), active coping (73.8%), and acceptance (76%). Conversely, the four least utilized mechanisms included behavioral disengagement (53.4%), denial (49.9%), venting (42%), and substance use (38.3%) (Supplementary Table 2).

### ProQoL and personal factors

This study explored the relationship between the ProQoL and personal factors. No statistically significant associations were observed between CS and the personal factors examined. However, males exhibited significantly higher levels of burnout (*p* = 0.004); older age and years of experience demonstrated a positive correlation with burnout (*r* = 0.437, *p* < 0.001; *r* = 0.349, *p* = 0.002, respectively). A statistically significant difference in education was noted regarding burnout (*p* = 0.025), particularly among graduates compared with university students. Additionally, being ever married (*p* = 0.049) and the nature of work (*p* = 0.049) were significant factors, as pairwise comparisons revealed that individuals involved in both office and fieldwork exhibited greater burnout levels than those solely engaged in fieldwork. However, for STS, males also exhibited significantly increased levels (*p* = 0.024). Additionally, a positive correlation was observed between age and years of experience in relation to burnout (*r* = 0.405, *p* < 0.001; *r* = 0.278, *p* = 0.014, respectively). Furthermore, a significant association was found between STS and previous mental diagnosis (*p* = 0.038), and between STS and shared trauma (*p* = 0.002) (Table 2).

**Table 2:** Professional quality of life and personal factors.

	CS	*p*	Burnout	*p*	STS	*p*
Sex						
Male	39.83 ± 6.0	0.697	26.57 ± 4.77	0.004*	30.34 ± 6.15	0.024*
Female	39.3 ± 5.83		23.23 ± 5.19		26.65 ± 7.66	
Age	*r* = 0.063	0.584	*r* = 0.437	<0.001*	*r* = 0.405	<0.001*
Education						
Secondary student	39.33 ± 5.76	0.919	25.25 ± 5.07	0.025*	27.08 ± 6.07	0.212
University student	39.85 ± 5.96		22.97 ± 4.83		27.15 ± 7.93	
Graduate	39.28 ± 6.0		26.41 ± 5.3^$^		30.0 ± 6.67	
Marital status						
Never married	38.82 ± 5.89	0.141	23.88 ± 5.02	0.049*	27.16 ± 7.16	0.052
Ever married	40.89 ± 5.71		26.33 ± 5.39		30.48 ± 6.95	
Nationality						
Single nationality	39.3 ± 5.89	0.181	24.7 ± 5.27	0.919	28.14 ± 7.38	0.353
Double nationality	43.0 ± 4.95		25.2 ± 5.36		30.80 ± 3.70	
Country of work						
Turkey	38.71 ± 6.0	0.136	23.96 ± 5.15	0.228	27.19 ± 6.88	0.103
Syria	41.59 ± 5.44		26.36 ± 5.65		31.32 ± 6.99	
Other	38.88 ± 5.59		24.88 ± 4.05		26.75 ± 8.29	
Nature of work						
Office work only	39.19 ± 5.73	0.364	24.61 ± 5.35	0.049*	27.81 ± 7.04	0.360
Field work and direct contact with victims only	38.28 ± 6.27		22.44 ± 3.93		29.76 ± 7.51	
Both office and field work	40.69 ± 5.78		26.28 ± 5.46^&^		28.31 ± 7.22	
Years of experience	*r* = 0.101	0.383	*r* = 0.349	0.002*	0.278	0.014*
Previous mental diagnosis						
Yes	41.5 ± 4.01	0.261	26.3 ± 5.5	0.314	32.7 ± 6.18	0.038*
No	39.25 ± 6.07		24.5 ± 5.21		27.66 ± 7.17	
Shared trauma						
Yes	39.92 ± 5.86	0.3	24.91 ± 5.26	0.656	29.49 ± 6.98	0.002*
No	38.0 ± 5.88		24.17 ± 5.39		22.83 ± 5.34	

The data are expressed as the mean ± SD, r; Pearson correlation ^$^significant with university students, ^&^significant with field work, ^*^Statistically significant *p* < 0.05.

Since no personal factors were significantly related to CS, we only performed a linear regression model between personal factors and burnout, and between personal factors and STS. The model for burnout was significant, with *F* = 3.259 and *p* = 0.003, and explained 27.7% of the variance; only graduation was the only predictor of burnout. For STS, the linear regression model was also significant (*F* = 9.669, *p* < 0.001) and explained a wide range of variability with *R*^2^ = 45.7%. Age, previous mental diagnosis and shared trauma predicted STS (Table 3).

**Table 3:** Linear regression model of personal factors predicting affecting professional quality of life (Model I).

Predictors	Unstandardized coefficients B std. error		Standardized coefficients Beta	*t*	Individual predictors sig	95% Confidence interval
Burnout^#^						
Constant	17.586	5.369		3.276	0.002*	6.874–28.299
Age	0.276	0.193	0.239	1.430	0.157	−0.109 – 0.662
Female^a^	−1.154	1.438	−0.110	−0.803	0.425	−4.023 – 1.715
Field work^b^	−1.562	1.562	−0.124	−1.000	0.321	−4.679 – 1.556
Office and field work^b^	0.418	1.432	0.039	0.292	0.771	−2.440 – 3.276
Ever married^c^	−0.736	1.390	−0.067	−0.530	0.598	−3.509 – 2.037
Education (secondary students)^d^	2.025	1.641	0.140	1.234	0.222	−1.250 – 5.299
Education (Graduate)^d^	3.033	1.259	0.285	2.408	0.019*	0.520–5.546
Years of experience	0.161	0.268	0.101	0.600	0.551	−0.375 – 0.697
Secondary traumatic stress^$^						
Constant	8.262	6.502		1.271	0.208	−4.705 – 21.229
Age	0.641	0.223	0.407	2.882	0.005*	0.197–1.085
Female^a^	−1.047	1.727	−0.073	−0.606	0.546	−4.491 – 2.397
Years of experience	−0.143	0.331	−0.066	−0.431	0.668	−0.802 – 0.517
Previous mental diagnosis^e^	4.506	2.100	0.213	2.146	0.035*	0.319–8.694
Shared trauma^e^	6.213	1.958	0.317	3.174	0.002*	2.308–10.117

^a^ref (Male), ^b^ref (other work nature), ^c^ref (never married), ^d^ref (other education), ^e^ref (no).

^#^*F* = 3.259, *p* = 0.003*, *R*^2^ = 27.7%, ^$^F = 6.542, *p* < 0.001*, *R^2^* = 31.8%.

### ProQOL and coping mechanisms

With respect to the correlation between ProQOL and coping mechanisms, among the three subscales, active coping exhibited a positive correlation with CS (*r* = 0.317, *p* = 0.005), and passive coping demonstrated a positive correlation with STS (*r* = 0.406, *p* = 0.001). Among the 14 coping mechanisms, statistically significant correlations with CS were found for planning (*r* = 0.354, *p* = 0.001) and acceptance (*r* = 0.279, *p* = 0.013). For burnout, significant positive correlations were found with venting (*r* = 0.257, *p* = 0.023) and substance use (*r* = 0.432, *p* < 0.001), while significant negative correlations were observed with acceptance (*r* = −0.231, *p* = 0.042) and religion (*r* = −0.399, *p* < 0.001). For STS, significant positive correlations were found with venting (*r* = 0.256, *p* = 0.023), denial (*r* = 0.299, *p* = 0.008), self-blame (*r* = 0.339, *p* = 0.002), substance use (*r* = 0.351, *p* = 0.002), and behavioral disengagement (*r* = 0.379, *p* = 0.001) (Table 4).

**Table 4:** Correlations between professional quality of life and coping mechanisms.

	Compassion satisfaction		Burnout		Secondary traumatic stress	
	*r*	*p*	*r*	*p*	*r*	*p*
General copinng mechanisms						
Active coping	0.317	0.005*	−0.100	0.384	0.130	0.255
Passive coping	−0.075	0.514	0.206	0.070	0.406	<0.001*
Seeking support	0.151	0.186	−0.043	0.705	0.158	0.166
Specific coping mechanisms						
Active coping	0.167	0.144	0.005	0.963	0.199	0.081
Informational support	0.180	0.115	−0.044	0.699	0.160	0.160
Positive reframing	0.080	0.484	0.047	0.684	0.180	0.115
Planning	0.354	0.001*	0.067	0.560	0.160	0.162
Emotional support	0.100	0.383	−0.036	0.754	0.133	0.247
Venting	0.130	0.256	0.257	0.023*	0.256	0.023*
Humor	−0.091	0.431	0.007	0.950	−0.073	0.525
Acceptance	0.279	0.013*	−0.231	0.042*	−0.221	0.052
Religion	0.208	0.068	−0.399	<0.001*	−0.052	0.652
Self-blame	0.026	0.824	0.114	0.319	0.339	0.002*
Self-distraction	0.009	0.938	−0.115	0.315	0.113	0.324
Denial	−0.120	0.296	0.046	0.687	0.299	0.008*
Substance use	0.054	0.636	0.432	<0.001*	0.351	0.002*
Behavioral disengagement	−0.069	0.551	0.204	0.074	0.379	0.001*

^*^Statistically significant *p* < 0.05.

According to the linear regression analysis of the general coping mechanisms, active coping emerged as a predictor of CS, whereas negative coping predicted both burnout and STS. In terms of specific coping mechanisms, planning was associated with a higher CS, substance use positively predicted burnout, and religion was a negative predictor. However, no specific coping mechanism was found to predict STS (Table 5).

**Table 5:** Linear regression model of coping mechanisms predicting professional quality of life (Model II, III).

Predictors	Unstandardized coefficients B std. error		Standardized coefficients Beta	*t*	Individual predictors sig	95% Confidence interval
Compassion satisfaction^&1,&2^						
Constant	27.235	5.526		4.928	<0.001*	16.223–38.246
Active coping	0.357	0.123	0.314	2.903	0.005*	0.112–0.603
Passive coping	−0.238	0.159	−0.175	−1.493	0.140	−0.555 – 0.079
Seeking support	0.351	0.216	0.190	1.626	0.108	−0.079 – 0.782
Constant	23.419	4.417		5.301	<0.001*	14.617–32.221
Planning	1.270	0.624	0.260	2.035	0.045*	0.026–2.513
Acceptance	0.516	0.487	0.134	1.058	0.294	−0.456 – 1.487
Religion	0.758	0.475	0.171	1.596	0.115	−0.188 – 1.704
Burnout^#1,#2^						
Constant	24.401	5.129		4.757	<0.001*	14.181–34.621
Active coping	−0.112	0.114	−0.110	−0.977	0.332	−0.339 – 0.116
Passive coping	0.326	0.148	0.268	2.203	0.031*	0.031–0.620
Seeking support	−0.225	0.200	−0.137	−1.123	0.265	−0.625 – 0.174
Constant	32.192	4.322		7.448	<0.001*	23.576–40.808
Venting	0.099	0.466	0.023	0.212	0.833	−0.830 – 1.028
Acceptance	−0.461	0.336	−0.134	−1.370	0.175	−1.131 – 0.210
Religion	−1.610	0.375	−0.407	−4.291	<0.001*	−2.358 – −0.862
Substance use	1.915	0.543	0.405	3.524	0.001*	0.832–2.999
Behavioral disengagement	−0.011	0.360	−0.003	−0.031	0.975	−0.729 – 0.706
Secondary traumatic stress^$1,$2^						
Constant	8.861	6.659		1.331	0.187*	−4.408 – 22.130
Active coping	0.136	0.148	0.097	0.916	0.363	−0.160 – 0.432
Passive coping	0.668	0.192	0.399	3.479	0.001*	0.285–1.050
Seeking support	−0.009	0.260	−0.004	−0.033	0.974	−0.527 – 0.510
Constant	10.369	5.638		1.839	0.070	−0.875 – 21.613
Active coping	1.071	0.608	0.195	1.763	0.082	−0.141 – 2.283
Venting	0.725	0.725	0.124	1.000	0.321	−0.721 – 2.172
Acceptance	−0.708	0.519	−0.150	−1.364	0.177	−1.742 – 0.327
Self-blame	0.846	0.859	0.124	0.985	0.328	−0.867 – 2.558
Denial	0.417	0.579	0.083	0.720	0.474	−0.738 – 1.573
Substance use	0.963	0.809	0.148	1.190	0.238	−0.651 – 2.577
Behavioral disengagement	1.070	0.660	0.221	1.620	0.110	−0.247 – 2.386

^&1^*F* = 4.065, *p* = 0.010*, *R*^2^ = 14.1%, ^&2^*F* = 4.875, *p* = 0.004*, *R*^2^ = 16.5%,^#1^*F* = 1.916, *p* = 0.134, *R*^2^ = 7.2%, ^#2^*F* = 8.586, *p* < 0.001*, *R*^2^ = 37.4%, ^$1^*F* = 5.190, *p* = 0.003*, *R*^2^ = 17.4%, ^$2^*F* = 4.419, *p* < 0.001*, *R*^2^ = 30.6%.

## Discussion

HAWs face various psychosocial stressors due to their work conditions and interactions with the traumatized individuals they aid, leading to indirect exposure to traumatic events. Consequently, many HAWs may experience distressing symptoms associated with personal factors or maladaptive coping mechanisms (Chen et al., 2020). Unmanaged CF might result in increased levels of absenteeism, impaired productivity, and work commitment, as well as complete cessation of aid work (Curling and Simmons, 2010). Therefore, studying CF, CS, and their association with other factors is crucial for improving the mental well-being of those working in the humanitarian aid field.

Despite most of our sample working in the Middle East, which is known for its geopolitical instabilities, the HAWs in our study exhibited high levels of CS. The absence of low CS levels has been reported in Hispanic caregivers serving Hispanic-displaced refugees in El Paso, Texas, a border region between the United States and Mexico (Lusk and Terrazas, 2015). This aligns with findings from other studies demonstrating elevated CS levels among professionals working with traumatized people (Burnett and Wahl, 2015; Craig and Sprang, 2010; Thieleman and Cacciatore, 2014). With respect to the CF subscales, our study revealed that nearly two-thirds of the participants exhibited a moderate level of burnout, while three-quarters demonstrated a moderate level of STS. Surprisingly, no participants reported high degrees of burnout, and only 1% exhibited high levels of STS. This could be justified by the high levels of CS, as CS is shown to negatively correlate with CF (Gonzalez-Mendez et al., 2020; Tessitore et al., 2023). Heightened CS may stem from the gratitude received from displaced people, a sense of accomplishment, and the valuable gained experience (Apostolidou, 2016). Working with displaced individuals can foster feelings of hope and inspiration, strengthen social connections, and enhance self-image (Barrington and Shakespeare-Finch, 2013).

In our study, male sex, older age, and more years of experience were associated with CF (both burnout and STS). These findings diverge from those of several studies on HAWs. A meta-analysis indicated that CF was either not associated with sex or was more prevalent among females (Cieslak et al., 2014). Other studies have suggested that older and more experienced workers exhibit lower levels of distress and CF, possibly due to their adoption of coping strategies or the quitting of individuals already experiencing significant distress.(Eriksson et al., 2009; Lopes Cardozo et al., 2005) Noticeably, some studies conducted on internal and external displaced refugees from Syria have indicated that men are often exposed to more traumatic events and are at a greater risk of developing PTSD (Tekeli-Yesil et al., 2018). This may imply differing gender roles in non-Western contexts, leading to variable exposures to traumatic experiences. Similarly, the observed associations in our study between CF and older age, male sex, and increased experience could be due to higher job responsibilities and increased workload (Hensel et al., 2015; Ogińska-Bulik et al., 2021).

This may imply differing gender roles in non-Western contexts, leading to variable prevalence rates for these issues. The observed correlations between older age, male sex, and increased experience with higher job responsibilities and workload could offer further insights into these associations.

A history of mental diagnosis was associated with STS in our study. Of the 41 possible variables, a systematic review revealed that a previous psychiatric diagnosis was one of three reliable predictors of psychiatric disorders, including secondary trauma and PTSD symptoms (Opie et al., 2020). A previous study proposed that this process is linked to adopting a negative coping mechanism (Foo et al., 2023).

In our study, shared trauma was significantly associated with higher STS scores and served as a predictor of STS according to linear regression analysis. Working in crisis situations renders HAWs both helpers and survivors themselves, exposing them to trauma directly and indirectly, and increasing their vulnerability to traumatic stress reactions (Lindsay, 2007; Tosone et al., 2011). Shared trauma involves a combination of symptoms of PTSD and STS due to the double exposure to trauma personally and professionally (Tosone et al., 2015), and such exposure makes it difficult to distinguish symptoms related to personal traumatic exposure from those related to professional work (Ehring et al., 2011). On the other hand, the qualitative analysis of shared exposure revealed that shared reality for workers with displaced individuals could be an additive advantage, as it creates a common background with regard to language and cultural barriers, allowing for more empathy and understanding, and in turn enhancing the levels of compassion satisfaction (Hamid et al., 2020). Another study reported that the experience of trauma could be a motive to work in the relief field as a source of meaning and life purpose (Wang et al., 2013).

The findings from our linear regression analysis revealed that heightened levels of active coping were indicative of greater CS, whereas increased negative coping was linked to higher levels of burnout and STS. For the specific coping mechanisms in the linear regression, planning demonstrated a positive correlation with CS; substance use was identified as a positive predictor of burnout, whereas religion emerged as a negative predictor. Religion and planning were the most prevalent coping mechanisms in the sample. Few studies have examined the coping mechanisms among HAWs, therefore, understanding the role of coping mechanisms in the lives of HAWs is crucial; in response to distress, HAWs use maladaptive coping mechanisms such as social isolation, anger, unhealthy eating, and alcohol or smoking (Jachens et al., 2016; Young et al., 2018).

Religion was the most prevalent coping mechanism among participants in this study. In the Middle East, religion and spirituality constitute central frameworks in people’s daily lives, serving as active components of identity and being widely utilized as a resource to overcome life difficulties (Koenig and Al Shohaib, 2023). These values highly prioritize charity and aiding those in need, underscoring the significance of compassion and altruism in religious teachings (Abo El Nasr and Eltaiba, 2016; Chaney and Church, 2017; Soliman, 2017). Religiosity and spirituality were found to be significant protective factors and linked to mental wellbeing, as perceived divine support can mitigate the effects of burnout, negatively correlate with different aspects of burnout, (Shin et al., 2014) and contribute to posttraumatic growth (Cardozo et al., 2013; Eriksson et al., 2015). These findings align with our results, in which religion exhibited a negative correlation with burnout. Although religion is perceived as a coping mechanism against stress, certain forms of religious coping are associated with adverse mental outcomes, including spiritual discontent, passive religious deferral, reassessment of God’s powers, and punitive God’s reappraisal (Ano and Vasconcelles, 2005; Grey et al., 2024). However, the Brief-COPE scale used in our study did not include distinctive items for assessing the different types of religious coping.

Planning was the second most prevalent coping mechanism and was positively linked to compassion satisfaction; it involves actively determining the best approach to address a stressor by considering appropriate actions to take as stress increases (Carver et al., 1989). It may serve as a strategy for those facing emergencies and are experiencing crisis situations regularly in their jobs (Yeşil and Polat, 2023). Such insights could be leveraged at the organizational level to establish effective plans for both organizational and personal growth. Substance use was the least-reported coping mechanism and was associated with higher rates of burnout. This is considered a form of passive coping. The low prevalence of substance use, such as alcohol use, among HAWs can be attributed to cultural beliefs (Lopes Cardozo et al., 2005).

Our study has some limitations. First, its cross-sectional design prevented the establishment of causal relationships. Second, the use of self-report questionnaires introduces the potential for recall or social desirability bias. Furthermore, the relatively small sample size and restriction to a single organization might limit the generalizability of our findings due to possible sampling bias and geographical restrictions. However, prior research has indicated that organizational type may not have a significant impact on mental health symptoms (Strohmeier and Scholte, 2015). Moreover, no specific data were collected on the nature of prior mental health diagnoses due to the sensitivity of the question and the relatively small sample size, which limited the potential for further analysis. This factor could be important in understanding predispositions to CF within our population. Therefore, further research with a larger sample is needed to explore the impact of mental health diagnoses on mental health outcomes in similar populations.

On the other hand, this study highlighted the frequency of CF and CS among a specific group of HAWs who work with displaced communities and shed light on the role of shared trauma and general and specific coping mechanisms in the prevalence of CS and CF. The study findings could be used for further training and education regarding the impact of CS and CF on mental well-being and working abilities. As recommended by the UNHCR (Antares Foundation, 2012), HAWs should be educated on alarming signs that require seeking mental health help, as many altruistic tendencies and a sense of obligation toward work might impede seeking help, thereby perpetuating CF (Gemignani and Giliberto, 2020). Organizations can contribute to empowering humanitarian aid workers (HAWs) by recognizing the threshold at which increased workload may lead to heightened mental distress (Posselt et al., 2020). Additionally, organizations can help provide training and education on the effective management of negative emotions and provide insights into the role of coping mechanisms, as previous psychological training is associated with less burnout, increased levels of well-being, and individual (Young and Pakenham, 2021). Moreover, organizations are advised to have an organizational approach to mental symptoms and to consider psychological wellbeing prior to recruiting workers (Shah et al., 2007).

### Further research

Given the preliminary nature of our findings, further studies with larger, more representative sample sizes across different organizations are needed. Future research should focus more on the nature of work and levels of responsibility in relation to mental health outcomes, as well as emphasize the role of shared trauma and different types of coping mechanisms among HAWs. We hope this study serves as a catalyst for longitudinal studies, inspiring more comprehensive research in this critical area.

## Conclusion

This study aimed to address key gaps in the literature about the psychology of HAWs in the Middle East. A significant frequency of CS and CF among HAWs supporting displaced communities. Factors such as sex, age, years of experience, and shared trauma were associated with burnout and STS. Specific coping mechanisms, such as religion and planning, positively impact HAWs’ mental well-being of HAWs, while substance use is linked to higher burnout rates. The authors have identified several recommendations including providing education on recognizing signs of mental distress among HAWs, implementing coherent organizational approaches to support their mental health, offering psychosocial training at the point of recruitment and at regular intervals thereafter, and fostering emotional caring and supportive leadership within aid organizations. Moreover, further research is needed to explore the role of shared trauma and coping mechanisms on the psychology of HAWs.

## Data Availability

The raw data supporting the conclusions of this article will be made available by the authors, without undue reservation.
