# Supplementary material for: Compassion satisfaction and compassion fatigue in humanitarian aid workers: the relationship with shared trauma and coping mechanisms
Source: Front Psychol. 2025 Apr 2;16:1522092. doi: 10.3389/fpsyg.2025.1522092 (PMC12000032; doi:10.3389/fpsyg.2025.1522092)
Supplement: Supplementary file 1 [file Supplementary_file_1.docx]

**Compassion Satisfaction and Compassion Fatigue in Humanitarian Aid Workers: The Relationship with Shared Trauma and Coping Mechanisms**

**Supplementary materials**

**Supplementary Table 1: General coping strategy scores of the studied sample**

|  | **Study sample (n= 78)** |
| --- | --- |
| **Active coping** |  |
| Min. – Max. | 30.0 – 48.0 |
| Mean ± SD. | 38.31 ± 5.17 |
| Mean% | 68.41% |
| **Passive coping** |  |
| Min. – Max. | 13.0 – 33.0 |
| Mean ± SD | 21.46 ± 4.31 |
| Mean% | 53.65% |
| **Seeking support** |  |
| Min. – Max. | 4.0 – 16.0 |
| Mean ± SD | 10.58 ± 3.18 |
| Mean% | 66.13% |

**Supplementary Table 2: Specific coping strategy scores of the studied sample**

|  | **Study sample (n= 78)** |
| --- | --- |
| **Active coping** |  |
| Min. – Max. | 2.0 – 8.0 |
| Mean ± SD. | 5.90 ± 1.32 |
| Mean% | 73.75% |
| **Informational support** |  |
| Min. – Max. | 2.0 – 8.0 |
| Mean ± SD. | 5.35 ± 1.71 |
| Mean% | 66.88% |
| **Positive reframing** |  |
| Min. – Max. | 2.0 – 7.0 |
| Mean ± SD. | 4.63 ± 1.28 |
| Mean% | 57.88% |
| **Planning** |  |
| Min. – Max. | 2.0 – 8.0 |
| Mean ± SD. | 6.22 ± 1.2 |
| Mean% | 77.75% |
| **Emotional support** |  |
| Min. – Max. | 2.0 – 8.0 |
| Mean ± SD. | 5.23 ± 1.73 |
| Mean% | 65.38% |
| **Venting** |  |
| Min. – Max. | 2.0 – 8.0 |
| Mean ± SD. | 3.36 ± 1.24 |
| Mean% | 42.0% |
| **Humor** |  |
| Min. – Max. | 2.0 – 8.0 |
| Mean ± SD. | 4.86 ± 1.39 |
| Mean% | 60.75% |
| **Acceptance** |  |
| Min. – Max. | 2.0 – 8.0 |
| Mean ± SD. | 6.08 ± 1.53 |
| Mean% | 76.0% |
| **Religion** |  |
| Min. – Max. | 2.0 – 8.0 |
| Mean ± SD. | 6.72 ± 1.33 |
| Mean% | 84.0% |
| **Self-blame** |  |
| Min. – Max. | 3.0 – 8.0 |
| Mean ± SD. | 5.09 ± 1.06 |
| Mean% | 63.63% |
| **Self-distraction** |  |
| Min. – Max. | 2.0 – 8.0 |
| Mean ± SD. | 5.6 ± 1.66 |
| Mean% | 70.0% |
| **Denial** |  |
| Min. – Max. | 2.0 – 7.0 |
| Mean ± SD. | 3.99 ± 1.44 |
| Mean% | 49.88% |
| **Substance use** |  |
| Min. – Max. | 2.0 – 6.0 |
| Mean ± SD. | 3.06 ± 1.11 |
| Mean% | 38.25% |
| **Behavioral disengagement** |  |
| Min. – Max. | 2.0 – 8.0 |
| Mean ± SD. | 4.27 ± 1.49 |
| Mean% | 53.38% |
